# A Double Monstrosity

**Published:** 1896-06

**Authors:** J. F. Johnson

**Affiliations:** Hiawassee, Ga.


### CORRESPONDENCE.

### A DOUBLE MONSTROSITY.

HIAWASSEE, GA., April 6, 1896.

March 27th I was called to see a woman who was said to be in labor. When I arrived I found her in labor, one foot protruding. On examination I found three other feet and legs not born. Soon I found the legs were all grown to one body. When the child was born it had four feet and legs well developed, four arms and hands; it had four ears and one face on the side of two heads. The body was grown together from the pubic bone up to the top of the head, making one united body. It had two well developed spinal columns. It was of neither sex; the umbilical cord came out just where the external organs should have been. It had two ani well developed; the umbilical cord was not over five or six inches in length; the placenta was grown or fully attached to the womb. The child was alive at birth, but died in a minute or two after birth. The mother of the child did well, and is reasonably healthy now. I tried very hard to get the parents to give me the child, but I could not get it. They would not even let me have its picture taken, and they buried the child at one corner of the garden for fear I would have it taken up. Many people went from Hiawassee to see the child. Now this is a very interesting case, one not often met with. I have never seen or read of one just like it.

J. F. JOHNSON, M.D.

### SIMPLE TEST FOR ALBUMIN.

Dr. A. C. Ewing (*Medical Record*) says: Draw up into a small glass pipette or tube about an inch of the urine, let the finger remain tightly over the top and insert the pipette into nitric acid and draw up under the urine about the same quantity of acid, when, if even a trace of true albumin be present, there will appear a beautiful line of demarkation between the acid and urine. This test is as accurate as it is simple and, besides, is decidedly economical and far less troublesome than all others.

